# Bid Regulates the Pathogenesis of Neurotropic Reovirus

**DOI:** 10.1371/journal.ppat.1000980

**Published:** 2010-07-01

**Authors:** Pranav Danthi, Andrea J. Pruijssers, Angela K. Berger, Geoffrey H. Holm, Sandra S. Zinkel, Terence S. Dermody

**Affiliations:** 1 Department of Biology, Indiana University, Bloomington, Indiana, United States of America; 2 Department of Pediatrics, Vanderbilt University School of Medicine, Nashville, Tennessee, United States of America; 3 Elizabeth B. Lamb Center for Pediatric Research, Vanderbilt University School of Medicine, Nashville, Tennessee, United States of America; 4 Department of Medicine, Vanderbilt University School of Medicine, Nashville, Tennessee, United States of America; 5 Department of Microbiology and Immunology, Vanderbilt University School of Medicine, Nashville, Tennessee, United States of America; University of North Carolina at Chapel Hill, United States of America

## Abstract

Reovirus infection leads to apoptosis in both cultured cells and the murine central nervous system (CNS). NF-κB-driven transcription of proapoptotic cellular genes is required for the effector phase of the apoptotic response. Although both extrinsic death-receptor signaling pathways and intrinsic pathways involving mitochondrial injury are implicated in reovirus-induced apoptosis, mechanisms by which either of these pathways are activated and their relationship to NF-κB signaling following reovirus infection are unknown. The proapoptotic Bcl-2 family member, Bid, is activated by proteolytic cleavage following reovirus infection. To understand how reovirus integrates host signaling circuits to induce apoptosis, we examined proapoptotic signaling following infection of Bid-deficient cells. Although reovirus growth was not affected by the absence of Bid, cells lacking Bid failed to undergo apoptosis. Furthermore, we found that NF-κB activation is required for Bid cleavage and subsequent proapoptotic signaling. To examine the functional significance of Bid-dependent apoptosis in reovirus disease, we monitored fatal encephalitis caused by reovirus in the presence and absence of Bid. Survival of Bid-deficient mice was significantly enhanced in comparison to wild-type mice following either peroral or intracranial inoculation of reovirus. Decreased reovirus virulence in Bid-null mice was accompanied by a reduction in viral yield. These findings define a role for NF-κB-dependent cleavage of Bid in the cell death program initiated by viral infection and link Bid to viral virulence.

## Author Summary

Viruses injure host tissues by activating signaling pathways that trigger cell death by a process called apoptosis. Hence, blockade of apoptosis may serve as a useful strategy to dampen the severity of viral disease. However, deployment of such a strategy requires identification of host signaling networks that control cell death and a detailed molecular blueprint of how these pathways are activated by a virus. In this study, we used mammalian reovirus, an important experimental model for studies of viral encephalitis, to elucidate how cell death pathways are activated following viral infection and whether these signaling cascades influence the capacity of a virus to produce lethal CNS disease. We found that Bid, a host regulator of cell death, influences apoptosis induction by reovirus. Moreover, Bid is required for efficient reovirus replication in the CNS and modulates reovirus neurological disease. These findings highlight Bid as a critical regulator of viral pathogenesis and illuminate a potential new target for development of antiviral therapeutics.

## Introduction

Tissue injury in response to infections by many viruses occurs as a consequence of apoptosis. Multiple studies using animal models of viral disease demonstrate a correlation between apoptotic potential and disease severity [1], [2], [3], [4]. These observations highlight proapoptotic signaling following virus infection as an attractive target for antiviral therapy. However, despite its central importance in viral pathogenesis, gaps in knowledge about the identity of death signaling pathways that modulate virus-induced apoptosis in vivo, along with an incomplete understanding of how these signaling cascades are activated during virus infection, have hampered the deployment of this strategy for treatment of viral disease.

Mammalian reoviruses injure infected cells via apoptosis both in culture and in tissues of infected animals. As such, studies of these viruses have contributed to an understanding of how virus infection culminates in apoptotic cell death. Unlike other viruses in which virulence correlates with cell-death capacity, the identity of viral and cellular factors that regulate reovirus-induced apoptosis in cell culture are for the most part known [4], [5], [6], [7], [8], [9], [10]. Moreover, many of these intermediaries also modulate reovirus-induced apoptosis in vivo [4], [7], [11], [12]. Studies using reassortant reoviruses [5], [6], ectopically expressed proteins [13], and genetically engineered reovirus mutants [4], [7] highlight a critical role for reovirus outer-capsid protein µ1 in apoptosis induction. Collectively, these studies indicate that prodeath signaling evoked by µ1 occurs subsequent to membrane penetration but prior to synthesis of viral RNA or protein [4], [7], [14], [15].

Classical death-receptor-mediated extrinsic apoptotic pathways stimulated by reovirus infection execute the death response [16]. Treatment of cells with soluble TRAIL receptors or expression of a dominant-negative form of Fas-associated death domain (FADD) protein blocks apoptosis, demonstrating that signaling via death receptors is required for execution of the apoptotic program [17]. In keeping with the function of extrinsic apoptotic signaling in reovirus infection, caspase-8 activation [18] and Bid cleavage [19] are observed in cells infected with reovirus [16]. Reovirus infection also stimulates intrinsic apoptotic pathways, as evidenced by release of cytochrome *c* and Smac/DIABLO from the mitochondria and activation of caspase-9 [16], [20], [21], [22], [23]. Concordantly, reovirus-induced apoptosis is dampened by over-expression of Bcl-2 [24], which inhibits mitochondrial apoptotic pathway activation [25].

Bid is a proapoptotic BH3-only member of the Bcl-2 family that functions to link the extrinsic apoptotic pathway and the mitochondrial amplification loop of the intrinsic pathway. Following death-receptor signaling, cytoplasmically resident Bid is cleaved by activated caspase-8 to generate a truncated form of Bid known as tBid [26]. tBid translocates to the mitochondria and triggers the release of cytochrome *c* and activation of the core mitochondrial apoptotic machinery [19], [27]. It is not known whether Bid plays a functional role in apoptosis induction by reovirus. Moreover, the relationship between apoptosis effector pathways and early events in viral replication are not understood.

In addition to these classical apoptotic pathways, the innate immune response transcription factor, NF-κB, is activated following reovirus infection [8]. NF-κB activation by reovirus depends on the viral µ1 protein and can be accomplished by genome-deficient reovirus particles [4], [7], [8]. Blockade of NF-κB signaling using chemical inhibitors or cell lines genetically deficient in NF-κB p50, NF-κB p65/RelA, IκB kinase (IKK)-α, or IKK adaptor IKKγ/NEMO significantly diminishes reovirus-induced apoptosis [8], [28]. Consistent with these findings, activation of NF-κB occurs within the first few hours of reovirus infection and precedes the biochemical and morphological hallmarks of apoptotic cell death [8], [28]. These observations suggest that NF-κB couples µ1-mediated events to the cellular apoptotic machinery. Although regulation and function of NF-κB has been extensively studied, the precise relationship between NF-κB and the cell-death machinery remains undefined.

In this study, we examined the function of cellular apoptosis regulator Bid using genetically deficient murine embryo fibroblasts (MEFs) and mice. We found that while Bid is dispensable for reovirus replication in cell culture, its function is required for reovirus-induced apoptosis. Blockade of NF-κB signaling, which diminishes apoptosis induction by reovirus [8], [28], prevents cleavage of Bid. In comparison to wild-type mice, Bid-deficient mice display diminished susceptibility to reovirus-induced CNS disease following either peroral (PO) or intracranial (IC) inoculation. Attenuated reovirus virulence in the absence of Bid is associated with decreased reovirus replication in the murine CNS. These results define an important role for Bid in virus-induced apoptosis and disease and illuminate Bid-dependent prodeath signaling as a viable target for antiviral therapy.

## Results

### Generation of tBid following reovirus infection is dependent on caspase-8

**Figure 1:**
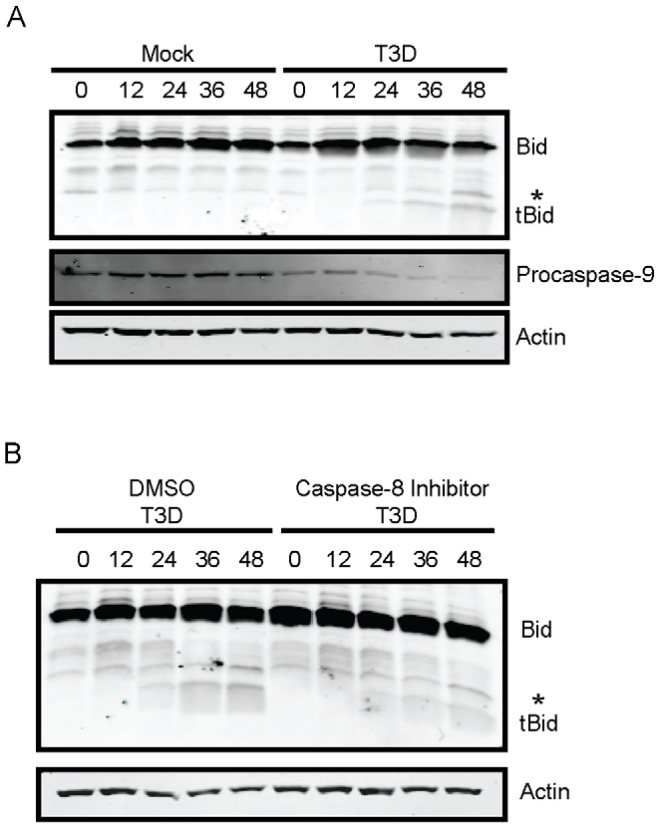
Bid is cleaved following reovirus infection of murine fibroblasts. (A) L929 cells were adsorbed with PBS (mock) or reovirus T3D at an MOI of 100 PFU/cell. Following incubation at 37°C for the indicated intervals, whole cell extracts were prepared, resolved by SDS-PAGE, and immunoblotted using antisera specific for Bid, actin, or procasapse-9. (B) L929 cells were adsorbed with reovirus T3D at an MOI of 100 PFU/cell. Following incubation at 37°C for the indicated intervals in the presence of 0 or 10 µM of Z-IETD-FMK, whole cell extracts were prepared, resolved by SDS-PAGE, and immunoblotted using antisera specific for Bid, actin, or procasapse-9. Protein bands are indicated on the right. A non-specific band is indicated by an asterisk (*).

Reovirus infection of HEK293 epithelial cells leads to a biphasic loss of full-length (FL) Bid [16]. Since a mitochondrial amplification loop through Bid is required for apoptosis only in some cell types, such as hepatocytes [29], [30], it is not known if Bid is cleaved in all cell types infected by reovirus. In addition, although calpains [31], [32], caspases [26], [33], [34], [35], and cathepsins [36], [37], [38], [39] can mediate Bid cleavage and have been implicated in apoptosis induction by reovirus [15], [16], [40], the precise identity of the protease that generates tBid following reovirus infection is not known. To determine whether tBid is generated following reovirus infection of fibroblasts, and to define the mechanism of Bid cleavage following reovirus infection, we infected murine L929 fibroblasts with reovirus strain type 3 Dearing (T3D) and monitored levels of FL Bid and tBid over 48 h (Figure 1A). While levels of FL Bid remained unchanged in mock-infected cells, we observed loss of FL Bid between 24 and 48 h post-infection. Decreased levels of FL Bid correlated with a corresponding increase in levels of tBid. To determine whether the generation of tBid results in activation of the mitochondrial loop of the intrinsic apoptotic pathway, we assessed levels of procaspase-9 as a surrogate for the formation of the caspase-9-containing apoptosome (Figure 1A). In a time frame consistent with cleavage-induced generation of tBid, we observed a decrease in procaspase-9 levels in reovirus-infected cells. These findings suggest that following reovirus infection of murine fibroblasts, Bid serves to activate the mitochondrial apoptotic pathway.

The adaptor molecule FADD is required for cleavage of Bid following reovirus infection of HEK293 cells [16]. Based on these data, we hypothesized that caspase-8 activity as a consequence of extrinsic prodeath signaling, is required for cleavage and activation of Bid. To test this hypothesis, we assessed the capacity of reovirus to mediate Bid cleavage in L929 cells treated with caspase-8 inhibitor Z-IETD-FMK (Figure 1B). As anticipated, Bid cleavage was not observed in mock-infected cells or mock-infected cells treated with Z-IETD-FMK (data not shown). Although tBid was generated at 36–48 h following reovirus infection of vehicle-treated cells, reovirus failed to efficiently induce activation of Bid in Z-IETD-FMK-treated cells until 48 h post-infection, providing evidence that reovirus evokes cleavage of Bid via caspase-8. In response to a variety of death agonists, Bid amplifies death signaling by linking the extrinsic (caspase-8) and intrinsic (caspase-9) apoptotic pathways [30]. Since our findings with reovirus parallel this pattern, our results suggest that Bid functions similarly following reovirus infection by linking the death-receptor and mitochondrial apoptotic pathways.

### Bid is required for reovirus-induced apoptosis

**Figure 2:**
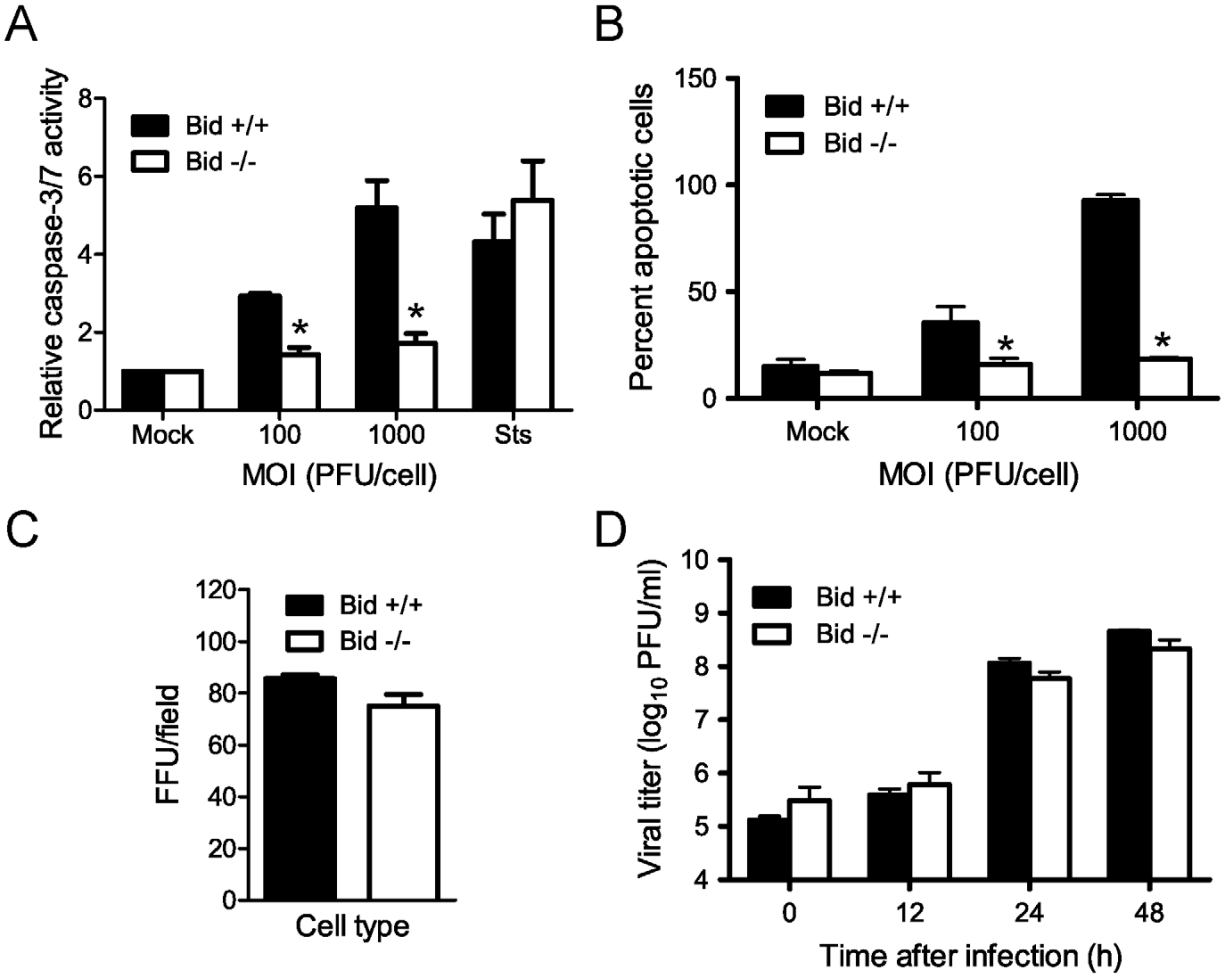
Bid is required for apoptosis induction following reovirus infection. (A) Wild-type or Bid-deficient MEFs were adsorbed with T3D at the MOIs shown. After incubation at 37°C for 24 h, caspase-3/7 activity in cell lysates was determined. Results are expressed as the mean ratio of caspase-3/7 activity from infected cell lysates to that from mock-infected cells for triplicate samples. Error bars indicate SD. *, *P*<0.05 as determined by Student's *t*-test relative to wild-type MEFs infected at an equivalent MOI. Cells were treated with 10 µM staurosporine (Sts) for 24 h as a control. (B) Wild-type or Bid-deficient MEFs were adsorbed with T3D at the MOIs shown. After incubation at 37°C for 48 h, cells were stained with AO. Results are expressed as the mean percentage of cells undergoing apoptosis for three independent experiments. Error bars indicate SD. *, *P*<0.05 as determined by Student's *t*-test relative to wild-type MEFs infected at an equivalent MOI. (C) Wild-type and Bid-deficient MEFs were adsorbed with 10^4^ particles/cell of T3D. After incubation at 37°C for 18 h, cells were visualized by immunostaining with polyclonal reovirus-specific antiserum, followed by incubation with Alexa546-labeled anti-rabbit IgG. Reovirus-infected cells were quantified by counting fluorescent cells. Results are expressed as mean fluorescent focus units (FFU) per field for triplicate samples. Error bars indicate SD. (D) Wild-type and Bid-deficient MEFs were adsorbed with T3D at an MOI of 2 PFU/cell. The inoculum was removed, and cells were incubated at 37°C for the times shown. Viral titers were determined after two cycles of freeze-thaw by plaque assay using L929 cells. Results are presented as mean titers from three independent experiments. Error bars indicate SD.

Signaling via the intrinsic pathway is essential for reovirus-induced apoptosis [16]. This observation, along with the dependence of mitochondrial apoptotic signaling on cleavage of Bid, suggests that Bid serves an essential function in reovirus-induced apoptosis. To directly test whether Bid is required for apoptosis induction following reovirus infection, we compared reovirus-induced apoptosis in wild-type and Bid-deficient MEFs. For these experiments, MEFs were infected with T3D, and apoptosis was assessed by chemiluminescent measurement of the activity of caspase-3 and caspase-7, which serve as effector caspases for both the extrinsic and intrinsic apoptotic pathways (Figure 2A). In comparison to mock-infected cells, infection of wild-type cells resulted in a significant increase in caspase-3/7 activity at 24 h post-infection. Since MEFs are poorly permissive for reovirus infection [41], staining of infected cells by indirect immunofluorescence indicated that adsorption with 100 PFU/cell of T3D resulted in infection of only ∼8% of cells at 20 h post infection (data not shown). Despite a low frequency of infection, this MOI resulted in an ∼3-fold increase in caspase-3/7 activity. When infection was initiated at 1000 PFU/cell, ∼20% cells were infected (data not shown), and caspase-3/7 activity increased ∼5-fold. In contrast, infection of Bid-deficient cells resulted in minimal caspase-3/7 activity following infection at either MOI. Increase in caspase-3/7 activity following treatment of each cell type with a broad-spectrum protein kinase inhibitor, staurosporine, was equivalent (∼5-fold), demonstrating that although Bid-deficient cells possess functional death-signaling pathways, they resist apoptosis induction by reovirus.

As an alternative means to quantify apoptosis, we compared wild-type and Bid-deficient MEFs for the onset of morphological characteristics of apoptosis following reovirus infection using an acridine orange (AO) staining assay (Figure 2B). Infection of wild-type cells resulted in a significant increase in the fraction of apoptotic cells at 48 h post-infection with 40% and 100% of the cells exhibiting apoptotic features at MOIs of 100 and 1000 PFU per cell, respectively. In contrast, Bid-deficient cells infected with T3D at either MOI displayed levels of apoptosis equivalent to mock-infected cells, ∼10%. Similar results were obtained following infection with another apoptosis-proficient reovirus strain, T3SA+ (data not shown). These data indicate that Bid is required for apoptosis induction following reovirus infection.

To determine whether decreased apoptosis in Bid-deficient cells is attributable to alterations in reovirus infection in the absence of Bid, we compared reovirus infectivity in wild-type and Bid-deficient cells using an indirect immunofluorescence staining assay (Figure 2C). An equivalent proportion of reovirus antigen-positive cells was detected at 20 h post-adsorption of wild-type and Bid-deficient cells. These data indicate that reovirus is capable of initiating infection in Bid-deficient cells. To determine whether reovirus completes a full infectious cycle in Bid-deficient cells, wild-type and Bid-deficient cells were adsorbed with T3D, and viral titers were determined by plaque assay at 0, 12, 24, and 48 h after infection (Figure 2D). Reovirus replicated with similar kinetics and produced equivalent yields in wild-type and Bid-deficient cells. Thus, the failure of Bid-deficient cells to undergo apoptosis in response to reovirus is not a consequence of diminished reovirus infection of these cells. We conclude that Bid is a key regulator of reovirus-induced apoptotic cell death.

### Signaling via NF-κB promotes cleavage-induced activation of Bid

**Figure 3:**
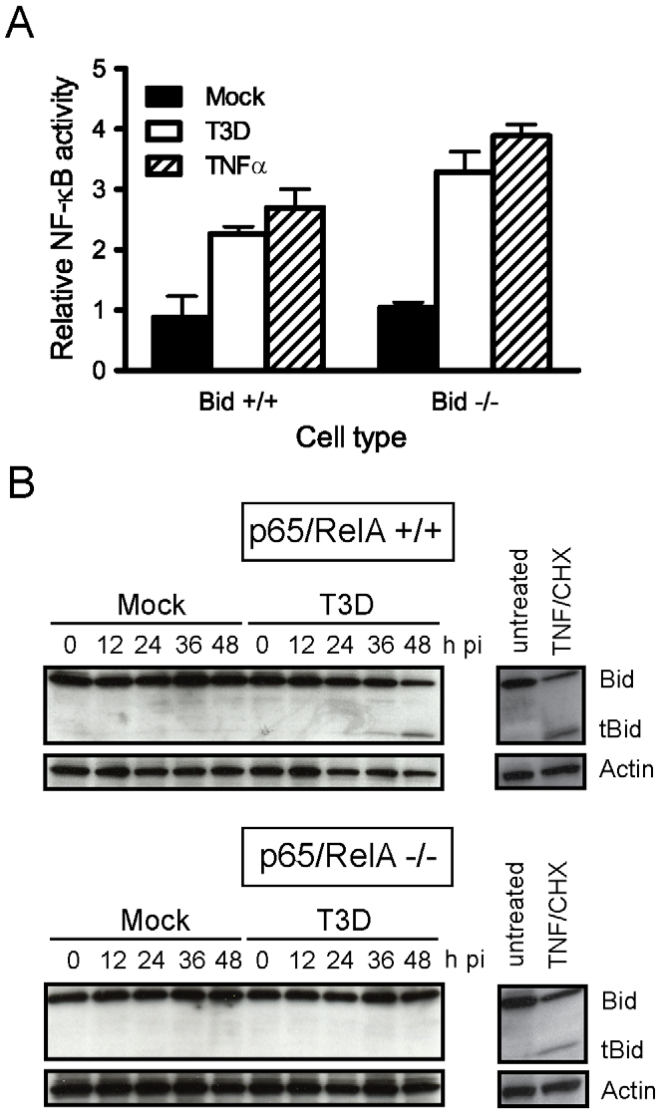
NF-κB is required for Bid cleavage following reovirus infection. (A) Wild-type MEFs or MEFs deficient in Bid were transfected with pNF-κB-luc, which expresses firefly luciferase under control of NF-κB, and a control plasmid, which constitutively expresses Renilla luciferase. At 18 h after transfection, cells were mock-infected or infected with T3D at an MOI of 100 PFU/cell. Cells treated with 10 ng/ml of TNFα were used as controls. Luciferase activity in cell lysates was quantified at 24 h following infection or 8 h following TNFα treatment. Results are expressed as the ratio of relative luciferase activity of experimental samples to relative luciferase activity of mock-infected cells. Error bars indicate SD. (B) Wild-type and p65/RelA-deficient MEFs were mock-infected, infected with T3D at an MOI of 100 PFU/cell, or treated with 10 ng/ml of TNFα and 10 µg/ml of cycloheximide (CHX). Whole cell lysates were prepared at the indicated times after infection or 12 h following TNFα/CHX treatment and resolved in 18% polyacrylamide gels and transferred to nitrocellulose membranes. The membranes were probed with polyclonal antisera specific for Bid or actin and appropriate HRP-conjugated secondary antibodies and visualized using chemiluminescence.

The identification of an essential role for Bid in apoptosis induction following reovirus infection allowed us to examine the relationship between NF-κB activation and Bid cleavage. To determine whether Bid is required for activation of NF-κB following reovirus infection, we compared reovirus-induced NF-κB activation in wild-type and Bid-deficient cells using a reporter assay. Wild-type and Bid-deficient MEFs were transfected with an NF-κB-luciferase reporter plasmid and infected with reovirus. Analogous to treatment with TNFα, a control NF-κB agonist, reovirus infection resulted in equivalent (∼2- to 3-fold) activation of NF-κB-driven gene expression in wild-type and Bid-deficient cells (Figure 3A). These results indicate that Bid is dispensable for NF-κB activation following reovirus infection and suggest that either reovirus-induced NF-κB activation occurs prior to Bid cleavage or that NF-κB activation and Bid cleavage occur in parallel but independent pathways that both function in apoptosis induction by reovirus.

To determine whether cleavage-induced Bid activation is dependent on NF-κB, we examined Bid cleavage in cells lacking p65/RelA, an NF-κB subunit required for apoptosis induction following reovirus infection [8]. Infection of wild-type MEFs with reovirus results in generation of tBid at 36–48 h after infection (Figure 3B). In contrast, infection of p65/RelA-deficient MEFs with reovirus did not lead to tBid generation even though efficient viral replication is observed in these cells [8]. Treatment of both wild-type and p65/RelA-deficient MEFs with apoptotic agonists TNFα and cycloheximide resulted in efficient cleavage of Bid, indicating that cell-death pathways leading to Bid cleavage are intact in both cell types. These findings suggest that cleavage and activation of Bid following reovirus infection requires NF-κB and place Bid cleavage subsequent to NF-κB signaling in response to reovirus infection. Moreover, since Bid amplifies death responses from the extrinsic apoptosis pathway by activating the mitochondrial loop, these findings suggest that death-receptor signaling during reovirus infection occurs in an NF-κB-dependent manner.

**Figure 4:**
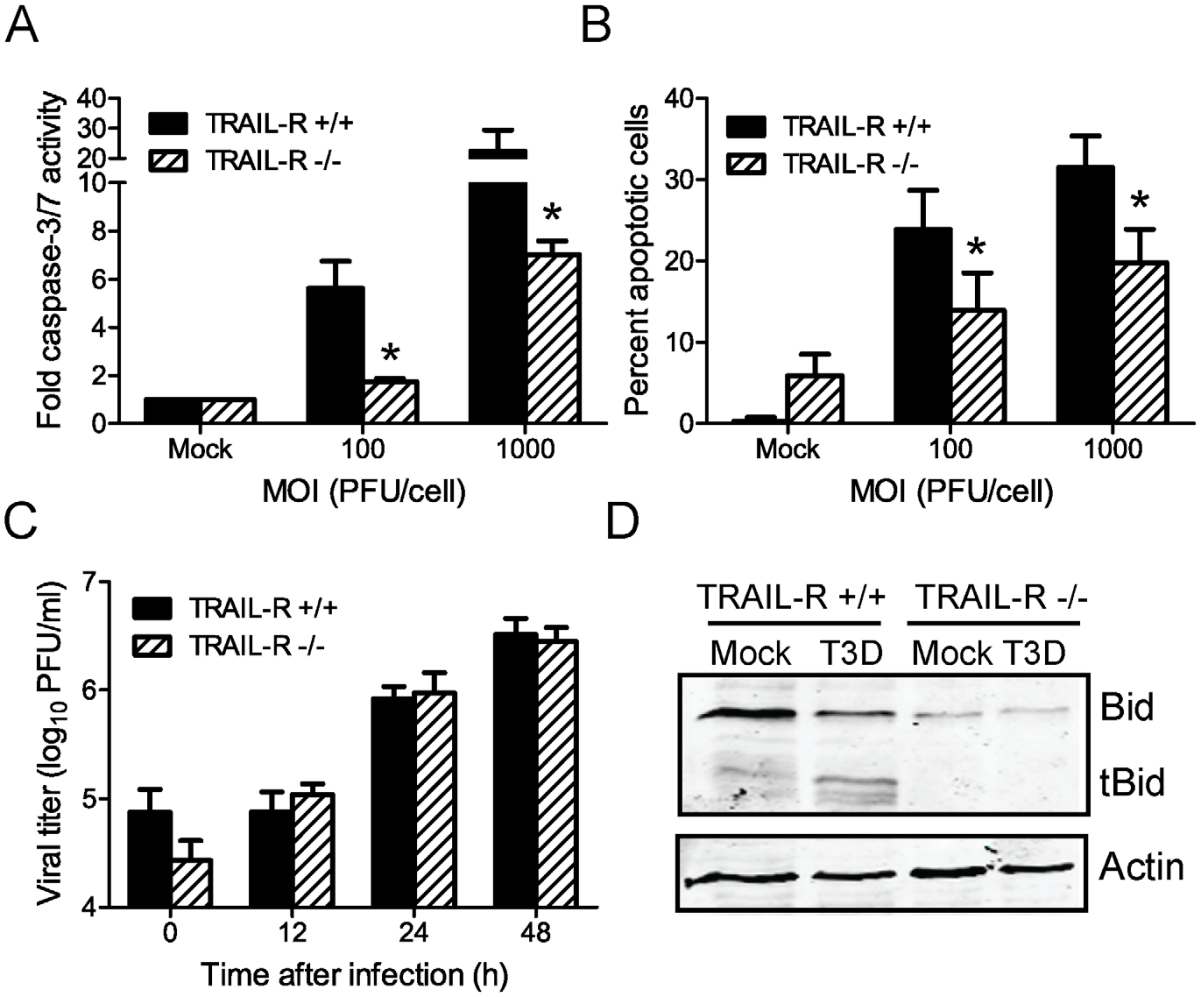
Death signaling via TRAIL-R contributes to Bid cleavage and apoptosis induction following reovirus infection. (A) Wild-type or TRAIL-R-deficient MEFs were adsorbed with T3D at the MOIs shown. After incubation at 37°C for 24 h, caspase-3/7 activity in cell lysates was determined. Results are expressed as the mean ratio of caspase-3/7 activity from infected cell lysates to that from mock-infected cell lysates for triplicate samples. Error bars indicate SD. *, *P*<0.05 as determined by Student's *t*-test relative to wild-type MEFs infected at an equivalent MOI. (B) Wild-type or TRAIL-R-deficient MEFs were adsorbed with T3D at the MOIs shown. After incubation at 37°C for 48 h, cells were stained with AO. Results are expressed as the mean percentage of cells undergoing apoptosis for three independent experiments. Error bars indicate SD. *, *P*<0.05 as determined by Student's *t*-test relative to wild-type MEFs infected at an equivalent MOI. (C) Wild-type and TRAIL-R-deficient MEFs were adsorbed with T3D at an MOI of 2 PFU/cell. The inoculum was removed, and cells were incubated at 37°C for 0, 12, 24 and 48 h. Viral titers were determined after two cycles of freeze-thaw by plaque assay using L929 cells. Results are presented as mean titers from three independent experiments. Error bars indicate SD. (D) Wild-type and TRAIL-R-deficient MEFs were mock-infected or infected with T3D at an MOI of 100 PFU/cell. Whole cell lysates were prepared at 48 h after infection and resolved in 18% polyacrylamide gels and transferred to nitrocellulose membranes. The membranes were probed with polyclonal antisera specific for Bid or actin and appropriate HRP-conjugated secondary antibodies and visualized using chemiluminescence.

Apoptosis-signaling pathways involving death receptors DR4 and DR5 and death ligand TRAIL, as well as Fas and FasL, have been implicated in apoptosis induction by reovirus [17], [42], [43]. However, it is not known which of these pathways mediates cleavage-induced activation of Bid. It is also not understood whether NF-κB regulates the activation of these pathways. Since upregulation of Fas following reovirus infection is dependent on prodeath signaling via c-Jun N terminal kinase (JNK) [43], and because JNK is activated via a mechanism distinct from NF-κB following reovirus infection [44], we focused our efforts on assessing the regulation and function of death-receptor signaling via TRAIL following reovirus infection. For these studies, we assessed the capacity of reovirus to induce apoptosis in MEFs lacking TRAIL-R, the only known receptor for TRAIL on murine cells [45], [46], [47] (Figure 4). In comparison to mock infection, T3D infection of wild-type cells resulted in an MOI-dependent ∼5- to 20-fold increase in caspase-3/7 activity at 24 h post-infection (Figure 4A). Although T3D infection of TRAIL-R-deficient cells also resulted in an increase in caspase-3/7 activity in comparison to mock-infection, the magnitude of this increase was only ∼2- to 6-fold. Assessment of apoptosis in wild-type and TRAIL-R-deficient MEFs using AO staining also showed an increase in apoptosis both in wild-type and TRAIL-R-deficient cells in comparison to mock-infected cells (Figure 4B). However, a substantially greater fraction of wild-type cells showed morphologic features of apoptosis in comparison to TRAIL-R-deficient cells infected at equivalent MOI, suggesting that efficient induction of apoptosis by reovirus requires TRAIL-R. T3D displayed comparable replication kinetics and produced equivalent yields in wild-type and TRAIL-R-deficient cells (Figure 4C). Thus, differences in the apoptotic potential of reovirus in wild-type and TRAIL-R-deficient cells are not associated with differences in reovirus growth in these cells.

To determine whether reovirus-induced cleavage of Bid is dependent on signaling via TRAIL-R, we monitored Bid cleavage following infection of TRAIL-R-deficient cells (Figure 4D). At 48 h post-infection of wild-type cells with T3D, FL Bid was cleaved to generate tBid. In contrast, FL Bid was not cleaved in T3D-infected TRAIL-R-deficient cells. While the apparent difference in the levels of FL Bid in wild-type and TRAIL-R-deficient cells was not reproducible, we consistently observed that levels of FL Bid remained unchanged in TRAIL-R-deficient cells following reovirus infection. These data indicate that TRAIL-R contributes to the induction of apoptosis by reovirus and suggest that cleavage of Bid following reovirus infection is dependent on TRAIL-R signaling.

### Reovirus displays attenuated virulence following PO inoculation of Bid-deficient mice

**Figure 5:**
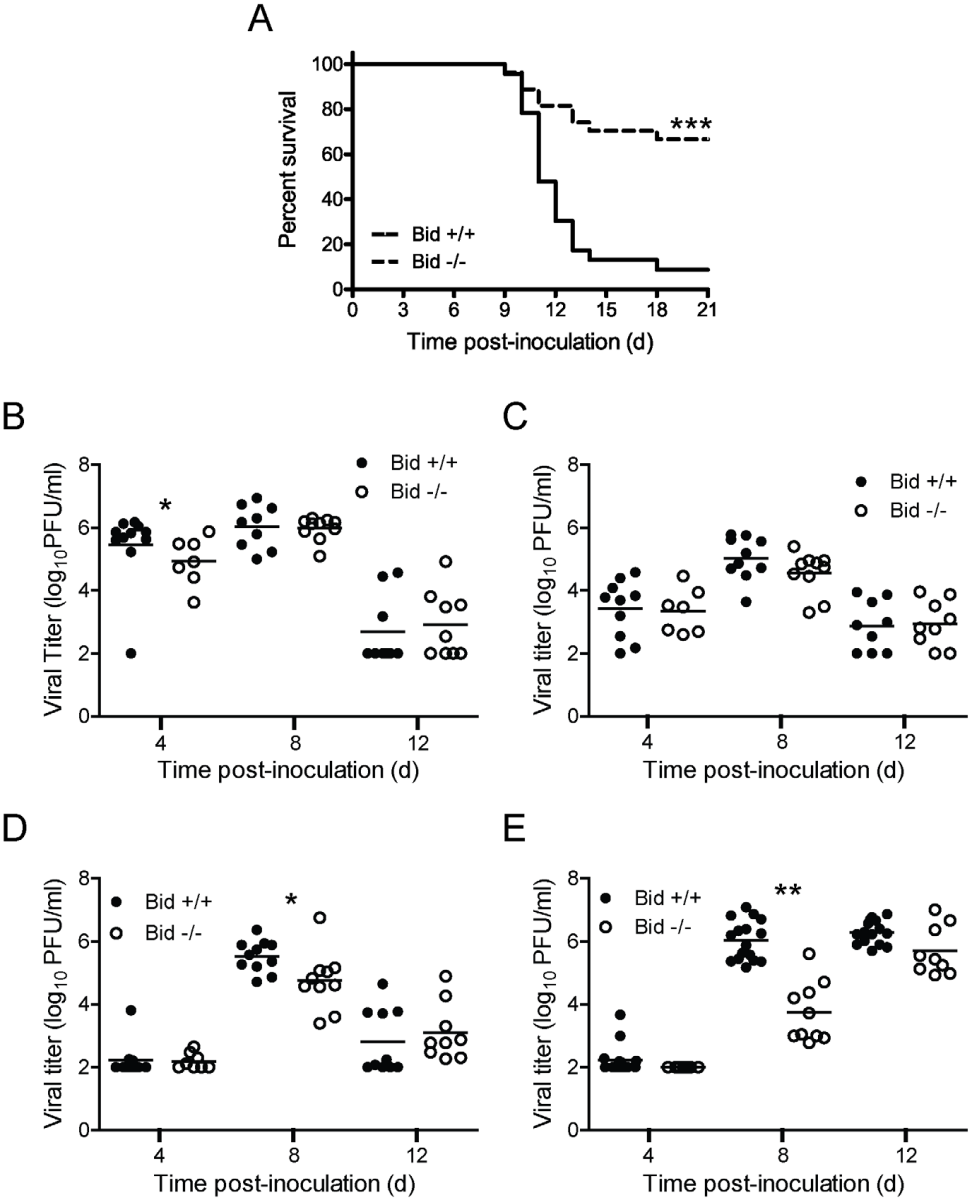
Bid modulates reovirus replication and pathogenesis following PO inoculation. (A) Two-day-old wild-type and Bid-deficient mice were inoculated perorally with 10^4^ PFU of T3SA+. Mice (*n* = 23 to 27) were monitored for survival for 21 days. ***, *P*<0.005 as determined by log-rank test in comparison to wild-type mice. (B–E) Two-day-old wild-type and Bid-deficient mice were inoculated perorally with 10^4^ PFU of T3SA+ and euthanized at the times shown. Intestines (B), liver (C), heart (D), and brain (E) were resected and homogenized by freeze-thaw and sonication. Viral titers in organ homogenates were determined by plaque assay. Results are expressed as viral titer in organs of single infected animals as indicated by closed (wild-type) or open (Bid-deficient) circles. Horizontal black lines indicate mean viral titers. *, *P*<0.05 as determined by Mann Whitney test in comparison to wild-type mice at the same time post-inoculation. **, *P*<0.01 as determined by Mann Whitney test in comparison to wild-type mice at the same time post-inoculation.

Reovirus virulence correlates with its capacity to cause apoptosis [4], [7], [11], [12], [48], [49]. Given the central role of Bid in apoptosis induction by reovirus in cell culture, we hypothesized that reovirus apoptosis and virulence would be diminished in the absence of Bid. To test this hypothesis, we inoculated two-day-old wild-type and Bid-deficient mice perorally with a highly virulent, enteric, neurotropic reovirus strain, T3SA+ [50], and monitored infected animals for signs of neurological disease and infection-induced morbidity over a period of 21 days (Figure 5A). Following inoculation with 10^4^ PFU of T3SA+, most wild-type mice developed paralysis and respiratory distress. In contrast, the majority of Bid-deficient mice were asymptomatic. Consistent with this observation, ∼91% of wild-type mice succumbed to reovirus infection with a median survival time of 11 days, whereas only ∼30% of Bid-deficient mice died. Due to the relative resistance of Bid-deficient mice to reovirus-induced encephalitis, a median survival time could not be determined. Thus, the cellular apoptotic regulator Bid modulates reovirus-induced encephalitis.

To determine whether the enhanced survival of Bid-deficient mice in comparison to wild-type mice following T3SA+ infection results from reduced reovirus replication, we compared titers of reovirus at sites of primary and secondary replication at 4, 8, and 12 d post-inoculation (Figure 5B–E). Peak titers of reovirus were comparable or slightly higher (∼5- to 10-fold) in the intestine, liver, and heart of wild-type mice in comparison to Bid-deficient animals. In contrast, substantially greater differences in peak reovirus titers were observed in the brain, with wild-type animals showing ∼25- to 100-fold higher titers in comparison to those in Bid-deficient mice at 8 d post-inoculation. However, by 12 d post-inoculation, titers of reovirus in wild-type and Bid-deficient mouse brains were equivalent. These findings suggest that reovirus infection is inefficient in the absence of Bid, especially in the CNS.

### Viral dose determines the requirement for Bid in reovirus encephalitis following direct IC inoculation

**Figure 6:**
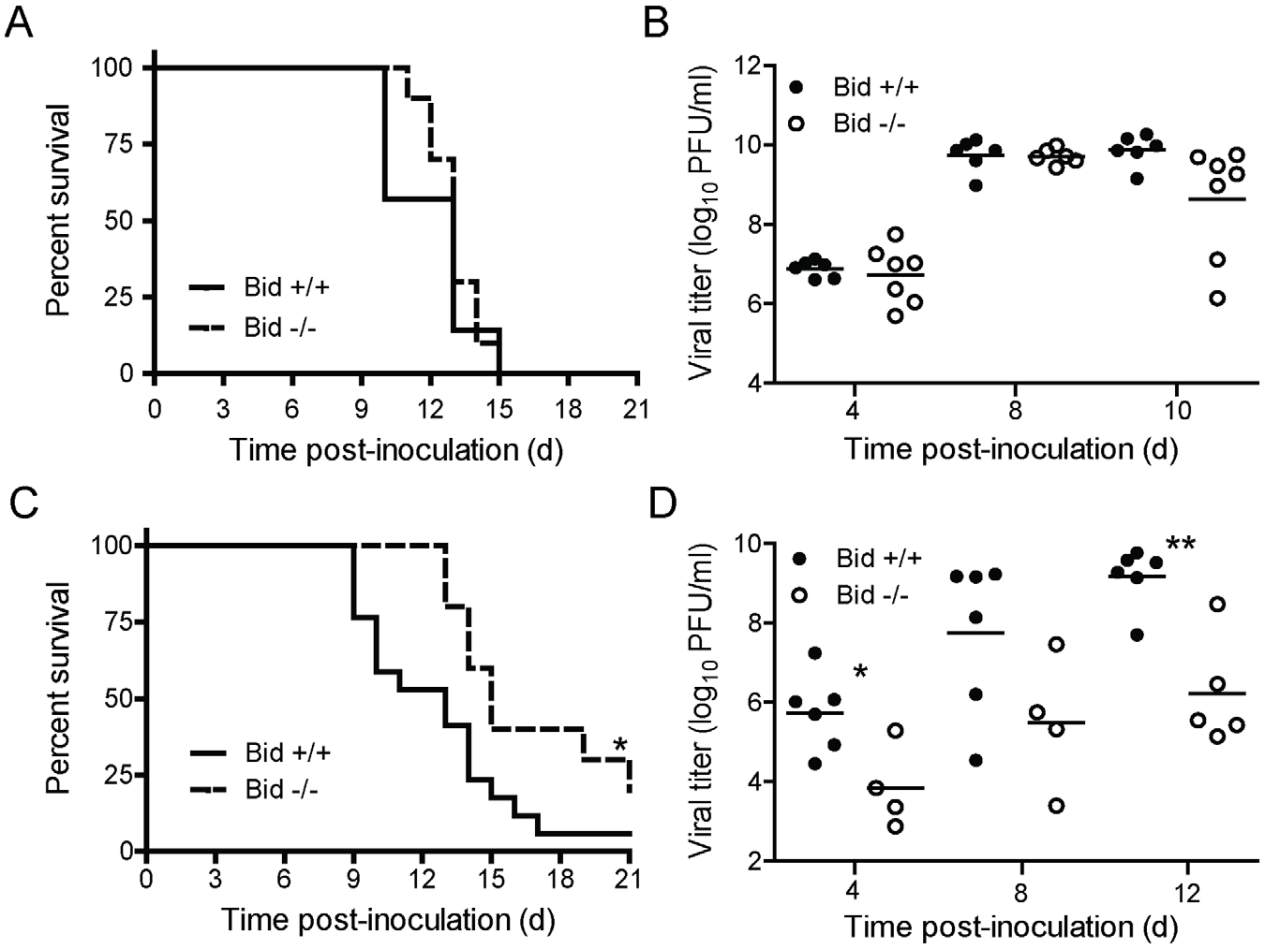
Bid modulates reovirus replication and pathogenesis following IC inoculation. (A and C) Two-day-old wild-type and Bid-deficient mice were inoculated intracranially with 100 PFU (A) or 5 PFU (C) of T3SA+. Mice (*n* = 13–23) were monitored for survival for 21 days. *, *P*<0.05 as determined by log-rank test in comparison to wild-type mice. (B and D) Two-day-old wild-type and Bid-deficient mice were inoculated intracranially with 100 PFU (B) or 5 PFU (D) of T3SA+ and euthanized at the times shown. Brains were resected and homogenized by freeze-thaw and sonication. Viral titers in brain homogenates were determined by plaque assay. Results are expressed as viral titer in organs of single infected animals as indicated by closed (wild-type) or open (Bid-deficient) circles. Horizontal black lines indicate mean viral titers. *, *P*<0.05 as determined by Mann Whitney test in comparison to wild-type mice at the same time post-inoculation. **, *P*<0.01 as determined by Mann Whitney test in comparison to wild-type mice at the same time post-inoculation.

Although titers of reovirus in the CNS were decreased in Bid-null mice following PO inoculation, it was not clear whether reduced reovirus titer in the CNS was a consequence of diminished reovirus dissemination to the CNS or diminished reovirus replication at that site. To distinguish between these possibilities, we inoculated wild-type and Bid-deficient mice intracranially with 100 PFU of T3SA+ and monitored infected animals for signs of CNS disease and mortality for 21 days (Figure 6A). At this dose of T3SA+, most wild-type and Bid-deficient mice displayed symptoms of neurological disease. Concordantly, both strains of mice succumbed to reovirus-induced disease with equivalent frequency and a median survival time of 13 days. Reovirus titers in the brains of wild-type and Bid-deficient mice also were comparable at 4, 8, and 10 d post-inoculation (Figure 6B). These results indicate that following a high-dose inoculation, Bid is dispensable for reovirus growth in the murine CNS and attendant encephalitis.

Peak titers of reovirus in the brains of intracranially-inoculated wild-type mice were ∼1000-fold higher than those in perorally-inoculated wild-type animals (compare Figures 5E and 6B). We thought it possible that this difference in viral load might contribute to the dramatic difference in the requirement for Bid in the pathogenesis of reovirus-induced CNS disease following PO and IC inoculation. To test this hypothesis, we inoculated wild-type and Bid-deficient animals intracranially with a considerably lower but still lethal dose of T3SA+, 5 PFU, and monitored infected animals for signs of reovirus encephalitis (Figure 6C). In comparison to wild-type mice in which ∼95% succumbed to disease, ∼70% of Bid-deficient mice developed lethal encephalitis. Moreover, the median survival time of wild-type mice infected with T3SA+ was significantly less (13 days) than that of Bid-deficient mice (15 days). To determine whether this difference in survival correlates with the efficiency of reovirus replication in the CNS, we compared titers of reovirus in brains resected from infected mice at 4, 8, and 12 d post-inoculation (Figure 6D). Titers of reovirus in brains of wild-type mice were substantially higher (∼10- to 100-fold) at each interval in comparison to those in Bid-deficient mice, with those at 4 and 12 d post-inoculation reaching statistical significance. These findings indicate that at a lower viral inoculum, Bid promotes efficient replication of reovirus in the CNS. Collectively, these data suggest that Bid influences reovirus virulence by regulating the growth of reovirus in the brain.

**Figure 7:**
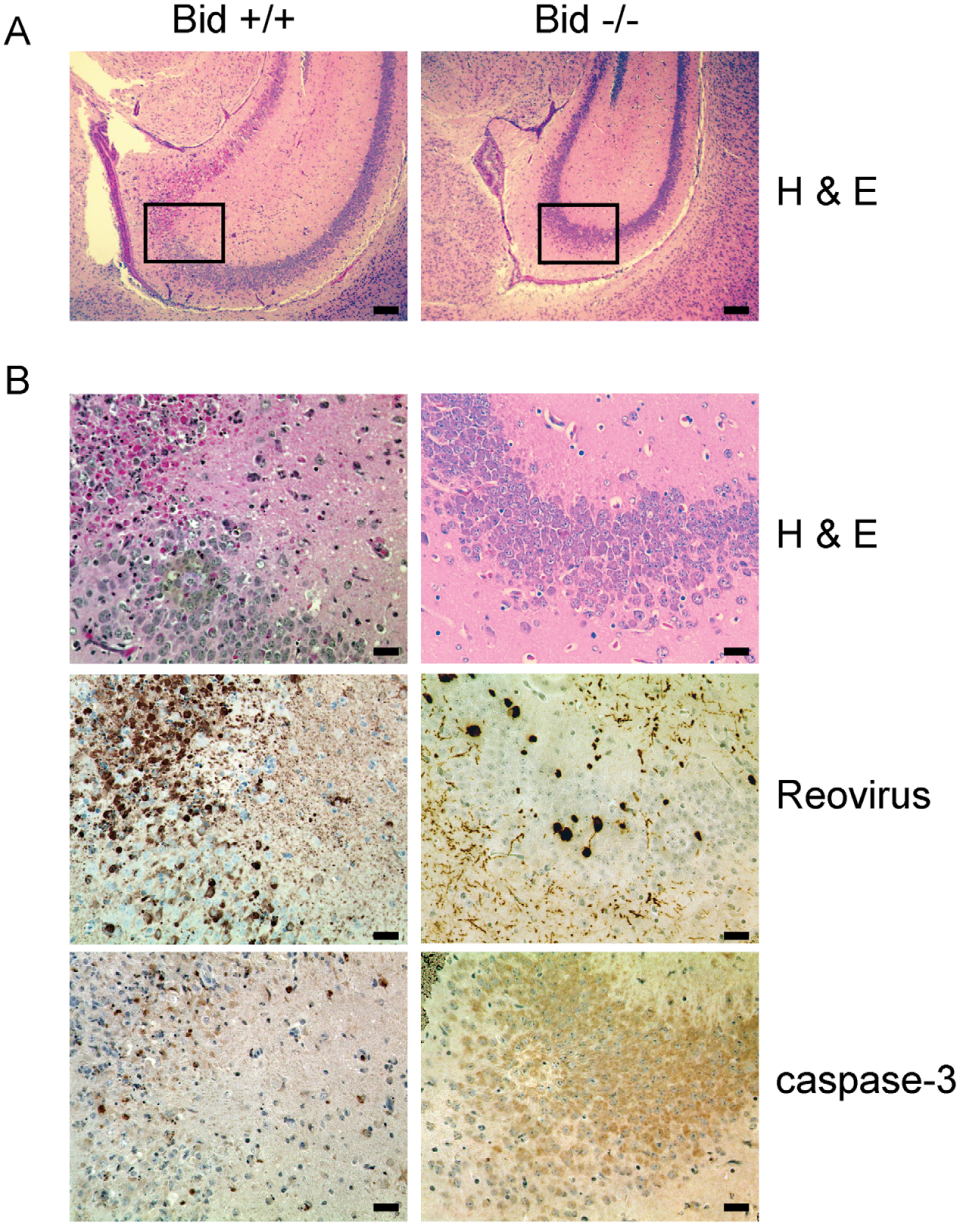
Reovirus-induced histopathologic injury is diminished in the CNS of Bid-deficient mice. Two-day-old wild-type and Bid-deficient mice were inoculated intracranially with 5 PFU of T3SA+. At 10 d post-inoculation, brains from infected mice were resected, fixed, and embedded in paraffin. (A) Low magnification image of H&E-stained hippocampal region of wild-type and Bid-deficient mouse brains. Scale bars, 500 µm. (B) Higher magnification images of the boxed region of the hippocampus shown in (A) stained in consecutive sections with H&E, polyclonal reovirus-specific antiserum, or activated caspase-3-specific antiserum. Scale bars, 100 µm. Intense brown staining indicates caspase-3- or reovirus-positive cells.

To assess the capacity of T3SA+ to produce neurological injury in the presence and absence of Bid, we examined hematoxylin and eosin (H&E)-stained coronal brain sections prepared from wild-type and Bid-deficient mice euthanized 10 d following IC inoculation with 5 PFU of T3SA+ (Figure 7). This time point was chosen to coincide with the presence of maximal viral titers following inoculation by this route. Since the inoculum used for these experiments was at least ∼10-fold lower than that used for most other studies of reovirus CNS pathogenesis [4], [7], [11], [12], [43], [48], [51], the extent of injury following infection of wild-type mice was not as extensive. Nonetheless, inoculation of wild-type mice with T3SA+ resulted in neuronal death in the cerebral cortex, hippocampus, thalamus, and hypothalamus, consistent with previous reports [4], [7], [11], [12], [43], [48], [51]. While the majority of infected wild-type mouse brains showed signs of injury, tissue damage was minimal in all of the brains examined from similarly infected Bid-deficient animals. Examination of the hippocampal region of a representative wild-type mouse brain at higher magnification showed damage to the CA3 region, with the pyramidal cells showing condensed nuclei characteristic of apoptosis (Figure 7B). In contrast, little damage was detected in an equivalent region of a Bid-deficient mouse brain (Figure 7B). These findings indicate that Bid is required for neurological injury produced by reovirus in mice.

To determine whether these differences in neurological injury are attributable to alterations in tropism of reovirus in the absence of Bid, sections of mouse brain were stained for reovirus antigen. Reovirus displayed similar tissue distribution in wild-type and Bid-deficient mouse brains, indicating that Bid expression does not influence reovirus tropism (data not shown). The CA3 region of a wild-type mouse brain showed reovirus antigen in areas coincident with extensive neuronal damage (Figure 7B). Regions positive for reovirus antigen also stained with an antibody for activated caspase-3. Although similar regions of Bid-deficient mouse brains contained reovirus antigen, staining was of diminished intensity and frequency (Figure 7B), consistent with the decreased efficiency of reovirus replication in the CNS (Figure 6B). Accordingly, few cells showing intense caspase-3 staining were observed in regions that contained reovirus. These data suggest that neuronal apoptosis following reovirus infection is diminished in the absence of Bid. Thus, Bid links reovirus replication and apoptosis induction in the production of fatal encephalitis.

## Discussion

Early steps in reovirus replication elicit apoptosis via a signaling pathway dependent on NF-κB [4], [7], [8], [14], [15]. It is not understood how virus-induced NF-κB activation leads to cell death. In this study, we evaluated the function and regulation of Bid in apoptosis caused by reovirus. We found that although Bid is dispensable for reovirus replication in cell culture, it is required for the induction of apoptotic cell death following reovirus infection. In this context, Bid is converted to its active, proapoptotic form, tBid, in an NF-κB-dependent manner. Generation of tBid in reovirus-infected cells requires signaling via TRAIL-R and caspase-8. These findings indicate that NF-κB signaling following reovirus infection results in activation of the extrinsic apoptotic pathway. In turn, the extrinsic apoptotic pathway evokes the mitochondrial apoptotic cascade via cleavage-induced activation of Bid. Together, these events culminate in the induction of apoptotic cell death.

Many viruses induce apoptosis via activation of host-encoded apoptosis-regulating factors. For example, the VSV M protein induces apoptosis by inhibiting the transcription of antiapoptotic factors such as Bcl-xl [52]. In other cases, virus-encoded polypeptides insert into mitochondrial membranes and trigger cytochrome *c* release, leading to activation of the mitochondrial apoptotic pathway. For example, influenza A virus PB1-F2 is thought to directly activate proapoptotic signaling by interaction with the mitochondrial membrane-associated factors ANT3 and VDAC1 [53]. Although one model for apoptosis induction by reovirus suggests that the φ fragment of reovirus µ1 protein induces apoptosis by directly targeting mitochondria analogous to PB1-F2 [13], our studies using apoptosis-defective reovirus mutants [4], [7], coupled with data presented here, support the idea that φ-mediated NF-κB signaling activates the mitochondrial apoptotic pathway indirectly via death-receptor signaling and Bid cleavage. This indirect mechanism of mitochondrial pathway activation by a proximal signal transducer also explains the timing of prodeath signaling in the reovirus replication cycle. We think that events associated with viral entry into cells, which are mediated by the µ1 φ fragment subsequent to membrane penetration, activate NF-κB within 1 h of infection [7], [28]. Unlike other NF-κB agonists such as TNFα which rapidly and transiently activate NF-κB, activation of NF-κB following reovirus infection is gradual and sustained and occurs maximally at 6–8 h post infection [28]. Activated NF-κB complexes lead to expression of genes that promote cleavage-induced activation of Bid at 24–36 h post infection and elicit characteristic features of apoptosis, including effector caspase activation and DNA fragmentation. These changes occur subsequent to completion of viral replication, and, therefore, apoptosis appears to have little detectable effect on viral growth in cell culture [4], [7], [8], [28]. Although unusual, NF-κB-dependent apoptotic pathways are also utilized by other viruses such as Dengue virus [54], HIV [55], infectious bursal disease virus [56], and Sindbis virus [57]. Thus, our studies may have uncovered a potentially conserved signaling pathway utilized by viruses to induce apoptosis via NF-κB.

It is not known how activation of NF-κB by reovirus culminates in cell death. Three previous studies have attempted to identify proapoptotic host genes that serve as effectors of the death response following reovirus infection. In the first, gene-expression profiles following infection with reovirus strains type 1 Lang (T1L) and type 3 Abney (T3A), which differ in the capacity to induce apoptosis [58], were compared by microarray analysis [59]. These experiments did not demonstrate differences in expression of death ligands or their respective receptors following infection by either strain. Thus, it was concluded that expression of these death mediators by reovirus is unlikely to contribute to apoptosis induction by reovirus. However, some differences were observed in expression of regulators of death-receptor signaling [59]. But since T1L and T3A display significant genetic diversity and vary in the modulation of multiple signaling pathways [9], [60], the contribution of NF-κB to the expression of prodeath genes could not be established. In the second study, gene-expression profiles following T3D infection in the presence and absence of functional NF-κB were compared [61]. Although this study identified several NF-κB-dependent genes that coordinate the cellular antiviral immune response, including numerous interferon-stimulated genes (ISGs), no classical components of death receptor-mediated signaling pathways or proapoptotic Bcl-2 family members were significantly upregulated in response to reovirus infection. In the third study, gene-expression profiles of reovirus strains that differ in the capacity to elicit translational shutoff were compared [62]. This study also demonstrated an increase in ISG expression but did not identify obvious NF-κB-dependent candidates that could serve to activate death receptor signaling.

We think there are three possibilities to explain why apoptosis-regulating, transcriptional targets of NF-κB, such as death ligands (e.g., FasL and TRAIL) [63], [64], death receptors (e.g., Fas and DR) [65], [66], and death-signaling regulators (e.g., Bax and Bcl-X_s_,) [67], were not identified in these studies. First, changes in the expression of prodeath genes activated by reovirus infection may be too transient to have been detected in the intervals selected for analysis. Second, the transformed nature of the cell lines used in these studies may not have been amenable to detection of alterations in gene expression induced by reovirus infection. Third, NF-κB activation following reovirus infection may regulate death signaling at a post-transcriptional level by an as yet unknown mechanism. In support of this idea, levels of DR5 protein increase following reovirus infection [17] but not its mRNA [59]. Additional studies using primary, non-transformed cell lines and genetically engineered viruses that differ only in the capacity to activate NF-κB are required to define how reovirus activates extrinsic apoptotic pathways to evoke cell death.

In addition to enhancing an understanding of mechanisms by which virus-induced signaling leads to activation of Bid, our studies highlight a critical role for Bid in controlling the pathogenesis of a viral disease. We found that Bid-deficient mice are less susceptible to lethal encephalitis produced by a neurotropic reovirus strain following either PO or IC inoculation. Reovirus replicates with slower kinetics in the absence of Bid, and virus-induced apoptosis and CNS injury are diminished in Bid-deficient animals. Although Bid contributes significantly to reovirus pathogenesis, our data do not allow us to determine whether diminished reovirus virulence in Bid-deficient animals is attributable to reduced capacity of reovirus to replicate in the CNS, diminished capacity of reovirus to injure neurons by apoptosis, or both effects. It is also not clear whether the decreased capacity of reovirus to evoke apoptosis in the CNS is a cause or effect of the lower viral titers at that site.

Because Bid serves to amplify the death response, it is not universally required for apoptosis induction. In some cell types, known as type I cells, caspase-8 activation results in direct, Bid-independent activation of the apoptosis effectors, caspase-3 and caspase-7 [29]. In others, known as type II cells, apoptosis requires amplification of death signals through stimulation of the mitochondrial pathway. In these cases, Bid serves to link the extrinsic and intrinsic apoptotic pathways [30]. Since the requirement for Bid in reovirus virulence is dependent on viral dose, we think that the role of Bid as an apoptosis regulator contributes to viral replication and consequent neurovirulence. Thus, we hypothesize that neurons function like type I cells when infected at a higher dose of virus and do not require the amplification of the mitochondrial apoptotic pathway via Bid to undergo apoptotic cell death. However, at lower infectious doses, neurons function like type II cells and require Bid-driven activation of the intrinsic mitochondrial apoptotic cascade to elicit cell death. This model also may explain why primary neuronal cultures infected with reovirus at a high MOI do not appear to require cytochrome *c* release and caspase-9 activation for apoptosis induction [42].

It is not known how Bid controls the efficiency of reovirus replication in the CNS. One possibility is that Bid-regulated apoptosis is required for efficient release of virus from neurons. Therefore, cell-to-cell spread of reovirus within the CNS may be inefficient in the absence of Bid. As an extension of this idea, blockade of apoptosis by other means also should cause a delay in reovirus replication. However, although symptoms of encephalitis are alleviated in NF-κB p50-deficient mice or in wild-type mice treated with a JNK inhibitor due to a reduction in virus-induced neuronal apoptosis, reovirus replication kinetics are not substantially diminished [11], [12]. Consistent with these findings, diminished virulence of apoptosis-defective reovirus mutants is not accompanied by significant decreases in reovirus replication efficiency in the CNS [4], [7]. Apoptosis following reovirus infection can occur in absence of p50, albeit at low efficiency [8]. Similarly, apoptosis-defective reovirus mutants retain some capacity to induce apoptotic cell death [4], [7]. Therefore, it is possible that in comparison to reovirus infection of Bid-deficient mice, CNS apoptosis was incompletely blocked in these other studies. Such a difference in the efficiency of apoptosis inhibition could explain the observed discrepancy in the requirement for Bid and other host or viral modulators of apoptosis for efficient replication of reovirus. A second possibility is that a Bid function not related to its capacity to regulate apoptosis contributes to reovirus replication in the CNS.

Analogous to its role in reovirus-induced cell death, Bid is implicated in apoptosis caused by many viruses [68], [69], [70], [71], [72], [73], [74], [75], [76], [77], [78], [79]. However, prior to our study, it was not known whether Bid modulates the pathogenesis of viral disease. The function of Bid in viral pathogenesis has been examined in a previous study, which found that the BH3-only protein, Puma, but not Bid, contributes to apoptosis-mediated elimination of antigen-specific T cells following acute infection with herpes simplex virus-1 [80]. Here, we demonstrate a pathogenic function for Bid in viral infection. Should Bid similarly modulate disease outcomes following infection by other virulent viruses, antiapoptotic compounds targeting Bid [81], [82], [83] may serve as useful antiviral therapeutics.

## Materials and Methods

### Cells and viruses

Murine L929 cells were maintained in Joklik's minimal essential medium supplemented to contain 10% fetal bovine serum (FBS), 2 mM L-glutamine, 100 U/ml penicillin, 100 µg/ml streptomycin, and 25 ng/ml amphotericin B (Invitrogen). Wild-type and Bid-deficient MEFs were maintained in Dulbecco's minimal essential medium (DMEM) supplemented to contain 10% FBS, 2 mM L-glutamine, 100 U/ml penicillin, 100 µg/ml streptomycin, and 25 ng/ml amphotericin B. TRAIL-R-deficient MEFs, prepared from D13 embryos, were maintained in DMEM supplemented to contain 10% FBS, 2 mM L-glutamine, 1× MEM nonessential amino acids, 0.1 mM 2-mercaptoethanol, 20 mM HEPES, 100 U/ml penicillin, 100 µg/ml streptomycin, and 25 ng/ml amphotericin B. Reovirus strain T3D is a laboratory stock. T3SA+ was generated by reassortment of reovirus strains T1L and type 3 clone 44-MA as described [84]. Purified reovirus virions were generated from second- or third-passage L-cell lysate stocks of twice-plaque-purified reovirus [85]. Viral particles were Freon-extracted from infected cell lysates, layered onto 1.2- to 1.4-g/cm^3^ CsCl gradients, and centrifuged at 62,000×*g* for 18 h. Bands corresponding to virions (1.36 g/cm^3^) were collected and dialyzed in virion-storage buffer (150 mM NaCl, 15 mM MgCl_2_, 10 mM Tris-HCl [pH 7.4]) [86].

### Antibodies and plasmids

Rabbit antisera raised against T1L and T3D have been described [87]. Rabbit antiserum specific for procaspase-9 was purchased from Cell Signaling. Goat antiserum specific for Bid was purchased from R & D systems, and goat antiserum specific for actin was purchased from Santa Cruz Biotechnology. HRP-conjugated anti-rabbit and anti-goat secondary antibodies were purchased from Amersham GE Biosciences. Alexa Fluor-conjugated anti-mouse immunoglobulin (Ig) G, anti-rabbit IgG, and anti-goat IgG secondary antibodies were purchased from Invitrogen.

Plasmids pRenilla-Luc and pNF-κB-Luc [88] were obtained from Dr. Dean Ballard (Vanderbilt University).

### Immunoblot assay

L929 cells or wild-type, Bid-deficient, or TRAIL-R-deficient MEFs were either adsorbed with reovirus at an MOI of 100 PFU/cell or mock-infected in serum-free medium at 4°C for 1 h, followed by incubation in serum-containing medium at 37°C for various intervals. Whole cell lysates were prepared by washing cells in phosphate-buffered saline (PBS) followed by lysis using 1× RIPA buffer (50 mM Tris [pH 7.5], 50 mM NaCl, 1% TX-100, 1% DOC, 0.1% SDS, and 1 mM EDTA) containing a protease inhibitor cocktail (Roche). Following centrifugation at 15,000×*g* to remove debris, the lysates were resolved by electrophoresis in polyacrylamide gels and transferred to nitrocellulose membranes. Membranes were blocked for at least 1 h in blocking buffer (PBS containing 5% milk or 2.5% BSA) and incubated with antisera against Bid (1∶1000), actin (1∶2000), or procaspase-9 (1∶1000) either at room temperature for 1 h or 4°C overnight. Membranes were washed three times for 10 min each with washing buffer (PBS containing 0.1% Tween-20) and incubated with1∶2000 dilution of horseradish peroxidase (HRP)-conjugated or Alexa Fluor-conjugated goat anti-rabbit Ig (for procaspase-9) or donkey anti-goat Ig (for Bid and actin) in blocking buffer. Following three washes, membranes were incubated for 1 min with chemiluminescent peroxidase substrate (Amersham Biosciences) and either exposed to film (for HRP-conjugated secondary antibodies) or scanned using an Odyssey Infrared Imager (LiCor).

### Assessment of caspase-3/7 activity

Wild-type, Bid-deficient, or TRAIL-R-deficient MEFs (10^4^) were seeded into black clear-bottom 96-well plates (Costar) and adsorbed with reovirus in serum-free medium at room temperature for 1 h. Following incubation of cells at 37°C for 24 h, caspase-3/7 activity was quantified using the Caspase-Glo-3/7 assay (Promega).

### Quantitation of apoptosis by AO staining

Wild-type, Bid-deficient, or TRAIL-R-deficient MEFs (5×10^4^) were grown in 24-well plates (Costar) and adsorbed with reovirus at room temperature for 1 h. The percentage of apoptotic cells after 48 h incubation was determined using AO staining as described [6]. For each experiment, >200 cells were counted, and the percentage of cells exhibiting condensed chromatin was determined by epi-illumination fluorescence microscopy using a fluorescein filter set (Zeiss Photomicroscope III; Thornwood, NY).

### Assessment of viral infectivity by indirect immunofluorescence

Wild-type or Bid-deficient cells (2×10^5^) were grown in 24-well plates and adsorbed with reovirus at room temperature for 1 h. Following removal of the inoculum, cells were washed with PBS and incubated in complete medium at 37°C for 18 h. Monolayers were fixed with methanol, washed twice with PBS, blocked with 2.5% Ig-free bovine serum albumin (Sigma-Aldrich) in PBS, and incubated successively for 1 h with polyclonal rabbit anti-reovirus serum at a 1∶1000 dilution and for 1 h with Alexa Fluor 546-labeled anti-rabbit IgG at a 1∶1000 dilution. Monolayers were washed with PBS, and infected cells were visualized by indirect immunofluorescence using a Zeiss Axiovert 200 fluorescence microscope. Reovirus antigen-positive cells were quantified by counting fluorescent cells in at least two random fields of view in triplicate wells at a magnification of 20×.

### Assessment of virus replication by plaque assay

Wild-type, Bid-deficient, or TRAIL-R-deficient MEFs (2×10^5^) in 24-well plates were adsorbed with reovirus at room temperature for 1 h in serum-free medium, washed once with PBS, and incubated in serum-containing medium for various intervals. Cells were frozen and thawed twice prior to determination of viral titer by plaque assay using L929 cells [89].

### Luciferase assays

Wild-type and Bid-deficient cells in 24-well plates were transfected with 0.72 µg/well of an NF-κB reporter plasmid, which expresses firefly luciferase under NF-κB control (pNF-κB-Luc), and 0.08 µg/well of control plasmid pRenilla-Luc, which expresses Renilla luciferase constitutively, using Fugene6 (Roche). After incubation for 24 h, transfected cells were adsorbed with reovirus in serum-free medium at room temperature for 1 h and incubated at 37°C in serum-containing medium for 24 h. Luciferase activity in the cultures was quantified using the Dual-Luciferase Assay Kit (Promega) according to the manufacturer's instructions.

### Infection of mice

Wild-type C57BL/6J mice were obtained from Jackson Laboratory. Bid-deficient mice backcrossed on to a C57BL/6J background for at least 8 generations have been previously described [30]. Two-day-old mice were inoculated either perorally or intracranially with purified virus diluted in PBS. PO inoculations were delivered in a volume of 50 µl by passage of a polyethylene catheter 0.61 mm in diameter (BD) through the esophagus and into the stomach [90]. The inoculum contained 0.3% (vol/vol) green food coloring to allow the accuracy of delivery to be judged. IC inoculations were delivered to the left cerebral hemisphere in a volume of 5 µl using a Hamilton syringe and a 30-gauge needle (BD Biosciences) [91]. For analysis of viral virulence, mice were monitored for weight loss and symptoms of disease for 21 days. For survival experiments, mice were euthanized when found to be moribund (defined by rapid or shallow breathing, lethargy, or paralysis). For determination of viral titer and immunohistochemical staining, mice were euthanized at various intervals following inoculation and organs were resected. For analysis of virus growth, organs were collected into 1 ml of PBS and homogenized by freezing, thawing, and sonication. Viral titers in organ homogenates were determined by plaque assay using L929 cells [89]. For immunohistochemical staining, organs were fixed overnight in 10% formalin, followed by incubation in 70% ethanol. Fixed organs were embedded in paraffin, and 5-µm histological sections were prepared. Consecutive sections were stained with H&E for evaluation of histopathologic changes or processed for immunohistochemical detection of reovirus antigens or activated caspase-3 [11]. Animal husbandry and experimental procedures were performed in accordance with Public Health Service policy and the recommendations of the Association for Assessment and Accreditation of Laboratory Animal Care and approved by the Vanderbilt University School of Medicine Institutional Animal Care and Use Committee.

## Supporting Information

Table S1 Comparative permissivity of L929 cells and MEFs to reovirus infection
(0.03 MB DOC)
