# Supplementary material for: Bid Regulates the Pathogenesis of Neurotropic Reovirus
Source: PLoS Pathog. 2010 Jul 1;6(7):e1000980. doi: 10.1371/journal.ppat.1000980 (PMC2895667; doi:10.1371/journal.ppat.1000980)
Supplement: Table S1 — Comparative permissivity of L929 cells and MEFs to reovirus infection (0.03 MB DOC) [file ppat.1000980.s001.doc]

Table S1: Comparative permissivity of L929 cells and MEFs to reovirus infection

| **Virus strain** | **Particle**^a^ **: PFU**^b^ | **Particle : FFU**^c^ | |
| --- | --- | --- | --- |
|  |  | L929 | MEF |
| T3D | 9.3 x 10^2^ | 4.6 x 10^3^ | 4.8 x 10^5^ |

^a^Viral concentration (in virions/ml) was determined by measuring absorbance of the purified viral preparation at 260 nm.

^b^Viral titer (in PFU/ml) was determined by plaque assay using L929 cells. Particle to PFU ratio was determined using the following formula: viral concentration/viral titer on L929 cells.

^c^Viral infectivity (in FFU/ml) was determined by indirect immunofluorescence using L929 cells or MEFs. Particle to FFU ratio was determined using the following formula: viral concentration/viral infectivity on L929 cells or MEFs.
